# Medication Consumption Patterns and Polypharmacy among Community-Dwelling Elderly in Lomé (Togo) in 2017

**DOI:** 10.1155/2020/4346035

**Published:** 2020-01-09

**Authors:** Fifonsi A. Gbeasor-Komlanvi, Wendpouiré I. C. Zida-Compaore, Ikpindi H. Dare, Aboudoulatif Diallo, Tchin P. Darre, Yao Potchoo, Mofou Belo, Didier K. Ekouevi

**Affiliations:** ^1^Faculté des Sciences de la Santé, Université de Lomé, Lomé, BP 1515, Togo; ^2^Centre Africain de Recherche en Epidémiologie et en Santé Publique, Lomé, BP 4089, Togo

## Abstract

**Background:**

In the sub-Saharan African, region of the world with a fast growing aging population and where the use of herbal products is very common, there is a paucity of data on medication consumption patterns among elderly people. The objective of this study was to assess the prevalence of polypharmacy and its associated factors among community-dwelling elderly in Lomé, Togo, in 2017.

**Methods:**

A cross-sectional study was conducted from March to June 2017 in Lomé, Togo among people aged 60 years and older. The Respondent Driven Sampling method was used to recruit participants. Data on socio-demographic characteristics and medication consumption patterns, including the use of medicinal plants and dietary supplements, were collected using a standardized questionnaire during a face-to-face interview at participants' home. Descriptive and binary logistic regression analyses were performed.

**Results:**

A total of 370 participants with median age 65 years, (IQR: 62–71) were enrolled in the study. Almost three elderly in five (57.6%) were multimorbid (had two or more chronic diseases). Conventional drugs (78.4%), medicinal plants (14.3%) and other dietary supplements (9.5%) were used by participants. The prevalence of polypharmacy was 22.7% (95% CI: 18.5–27.3%). Concurrent use of conventional drugs and medicinal plants or other dietary supplements was observed among 17.0% of participants and 67.3% reported self-medication. Multimorbidity (aOR = 4.55; 95% CI: [2.42–8.54]) and female sex (aOR = 1.86; 95% CI: [1.00–3.47]) were associated with polypharmacy.

**Conclusion:**

One elderly in five uses five or more medications in Togo. Further studies are needed to assess drug-drug interactions and herb-drug interactions among this population.

## 1. Introduction

Polypharmacy is defined as “the administration of many drugs simultaneously or by the administration of an excessive number of drugs” [1]. Also defined as the use of five or more medications daily, polypharmacy is more common among elderly people who tend to have more chronic medical conditions and have to use several medications [1, 2]. Although they are usually left out of clinical trials, elderly people are reported to have an important consumption of pharmaceutical drugs. In the United States, while accounting for less than 13% of the general population, it is estimated that 33% of annual prescriptions are for people aged 60 years and older [3]. As populations around the world are rapidly ageing, and the burden of chronic diseases is growing, polypharmacy is expected to rise significantly.

Although polypharmacy can be necessary to treat elderly people, it represents an important public health concern in this population. Several studies have reported that more than 50% of the elderly use unnecessary medications which are defined as medications with no indication, lack of effectiveness or therapeutic duplication [4, 5]. Commonly used products are prescribed medications but also nonprescribed products, including herbal products, vitamins and minerals and the concomitant use of these products may increase the risk of drug-drug interactions and herb-drug interactions [5, 6]. In a study conducted among nursing homes residents, Field et al., reported that taking more than 4 medications was associated with adverse drug events [7]. Indeed, the process of ageing involves a continuum of physiological changes which affect drug metabolism in the body, increasing the risk of adverse drug events and death [2, 4, 8]. Moreover, elderly people can have difficulty following complex treatments due to cognitive or visual impairment [1]. Finally, polypharmacy induces high medical costs stemming from direct drug costs and healthcare service utilization [2, 5, 8].

In sub-Saharan Africa, region of the world with a fast-growing aging population and an increasing burden of chronic diseases [9, 10], self-medication [11–13] and the use of medicinal plants are very common [14, 15]. However, data on medication consumption patterns among elderly people are rare. In addition, the very few studies which have examined polypharmacy among the elderly, were only focused on the use of conventional drugs [16, 17]. In Togo, life expectancy has increased from 40 to 60 years between 1960 and 2016 [18], but there is no geriatric services and data on elderly health status are scarce. The objective of this study was to assess the prevalence of polypharmacy and its associated factors among community-dwelling elderly in Lomé, Togo, in 2017.

## 2. Materials and Methods

### 2.1. Study Design and Population

A cross-sectional study was carried out from March to June 2017 in Lomé, capital city of Togo in West Africa.

Elderly people were recruited using the Respondent Driven Sampling (RDS) method [19, 20]. First, we randomly selected 62 community pharmacies among 117 registered community pharmacies in Lomé and authorization to conduct the study was obtained from 53 pharmacists. In each pharmacy, on a selected day, all the people who came to the pharmacy to buy a product or to be counselled, were approached by medical/pharmacy students. One or two of those aged 60 years and older were asked to participate in the study. Of the 78 elderly who were approached, 6 refused to participate in the study. Thus, a total of 72 “seeds” or “primary elderly persons” were recruited when they visited one of the participating community pharmacies. Each recruited participant was asked to refer three other elderly people until the required sample size was reached. A home visit was subsequently scheduled with recruited elders. Caregivers or family members who assist the elderly were solicited to give responses regarding the treatment used by the elderly.

Since no data on polypharmacy were available in Togo, the sample size calculation was based on the following assumptions: an expected prevalence of polypharmacy in people aged 60 years and older of 20% based on the estimate (19.7%) reported in a study conducted in Ethiopia [16], with a precision of 5%, a significance level set at 5%, and a nonresponse rate of 10%; the minimum sample size was estimated at 271 participants.

### 2.2. Data Collection

Participants were asked to show the investigators all products they were currently using for their treatment. For the present study, we used the term “medication” to refer to conventional drug (including prescription and over-the-counter or OTC drugs), medicinal plant/herbal product and dietary supplement (vitamins, minerals). Thus, medication use was defined as taking on a regular schedule (every day or at least once a week) conventional drug, medicinal plant/herbal product and dietary supplement. Medications used on a monthly basis were not included. Pro re nata (PRN) drugs were counted if at least 50% of daily recommended doses were taken by participants. For combination medications, each active pharmaceutical ingredient or compound was counted as one medication.

A 20-minute standardized questionnaire was administered to selected elderly during a face-to-face interview by sixty trained medical and pharmacy students at participants' home. Information collected included data on the respondents' socio-demographic characteristics (sex, age, education level, marital status, and monthly revenue), past medical history, and patterns of medication use: number, type, storage and management of medications, medications obtained with and without prescription.

### 2.3. Definition of Variables

Polypharmacy was the main variable of interest and was defined as the use of five or more medications on the day of the week when the number of medications scheduled for use is the highest [21]. We constructed two variables for polypharmacy: “polypharmacy 1” was defined as the use of five or more medications, only considering conventional drugs; and “polypharmacy 2” which was defined as the use of five or more products, including conventional drugs, medicinal plants and other dietary supplements. Excessive polypharmacy was defined as the use of 10 or more products, including conventional drugs, medicinal plants and other dietary supplements [22–24]. Self-medication was defined as the selection and use of medicines (prescription drugs or OTC) by individuals to treat self-recognized illnesses or symptoms within the week preceding the study. Concurrent use was defined as the concomitant use of conventional drugs and medicinal plants and / or other dietary supplements [6, 25].

### 2.4. Statistical Analysis

Descriptive statistics were performed and results were presented with frequency tabulations and percentages for categorical variables. Quantitative variables were presented as medians with their interquartile range (IQR). The prevalence was estimated with corresponding 95% confidence interval (95% CI). Binary logistic regression analyses were performed to identify factors associated with “polypharmacy 1” and “polypharmacy 2”. In the univariate logistic regression, variable with a *p*-value <0.20 were included into the multivariable analyses. A backward procedure approach was performed for variable selection and adjusted odds ratio (aOR) were reported with their 95% confidence interval (CI). All analyses were performed using Stata® software version 14 (College Station, Texas, TX, USA). The significance level was set at 5%.

## 3. Results

### 3.1. Socio-Demographic Characteristics and Past Medical History

A total of 370 participants with a median age of 65 years, (IQR: 62–71) were enrolled in the study. Female respondents represented 54.9% of the study population and they were more likely to be living alone, have no education level, and have lower monthly revenue compare with their male counterparts (*p* < 0.001) (Table 1).

The main medical conditions reported were osteoarthritis (59.5%), hypertension (50.7%), diabetes (18.2%), and visual impairment (13.0%) (Table 1). The prevalence of multimorbidity (≥2 chronic conditions) was 57.6% (95% CI: 52.4–62.7), and it was more common in female (63.0%) than in male elderly (57.5%) (*p* = 0.033).

### 3.2. Storage and Management of Medications

Medications were left out on a table (25.4%) or stored in plastic bags (54.9%) or in boxes (19.7%). Elderly's medications were stored in family-shared container for 14.1% of participants. Twenty-four (7.0%) elderly had expired medications. The majority of study participants reported storing (81.1%) and taking (83.8%) their treatments without the assistance of a caregiver.

### 3.3. Medication Consumption Patterns

**Figure 1:**
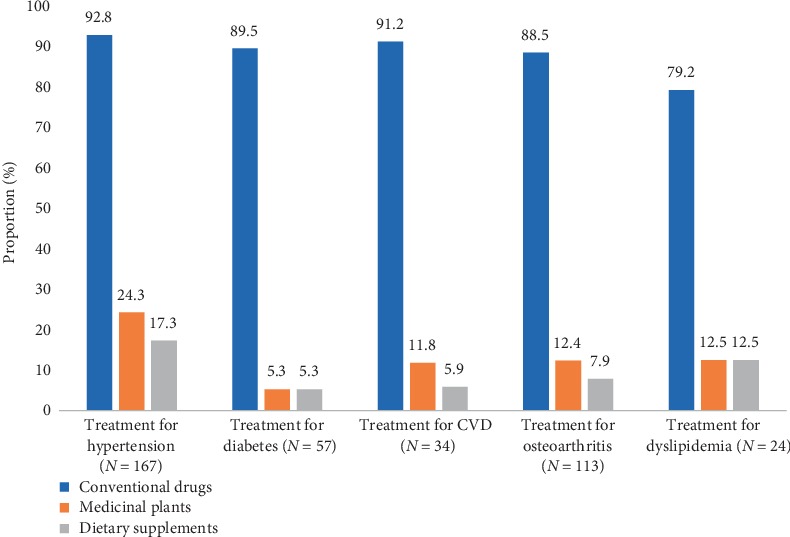
Types of medications used according to medical conditions. CVD: Other cardiovascular diseases.

Among recruited elderly, 18.6% were not taking any medication (including conventional drug, medicinal plant/herbal product and dietary supplement) and 81.4% took at least one medication (Table 2). The median number of medications taken daily was 2 (IQR: 2-3) for men and 3 (IQR: 1–5) for women (*p* = 0.074). Conventional drugs (78.4%), medicinal plants (14.3%) and other dietary supplements (9.5%) were used by participants. *Moringa olifeira* (18.8%) and *Allium sativum* commonly called “garlic” (9.4%) were the medicinal plants mostly found at elderly's home. For any of the main reported medical conditions, conventional drugs were the most used followed by medicinal plants (Figure 1). Concurrent use was observed among 63 (17.0%) participants.

The prevalence of self-medication was 67.3% (95% CI: 62.3–70.1) with no difference according to gender (69.5% among male vs 65.5% among female; *p* = 0.518) (Table 2). Drugs used for self-medication were obtained in community pharmacy (70.3%), from street vendors (39.4%), and from family and friends (18.1%).

For each chronic condition, the proportion of older adults using conventional drugs, medicinal plants, and other dietary supplements is presented in blue, orange, and grey bars, respectively.

#### 3.3.1. Prevalence of Polypharmacy

The prevalence of “polypharmacy 1” and “polypharmacy 2” was 17.0% (95% CI: 13.3–21.3%) and 22.7% (95% CI: 18.5–27.3%), respectively. Polypharmacy was higher in female than in male participants for “polypharmacy 1” (*p* = 0.210) and “polypharmacy 2” (*p* = 0.049) (Table 3). “Polypharmacy 2” was more frequent among participants who reported having hypertension, diabetes, osteoarthritis, dyslipidemia, visual impairment, and other cardiovascular diseases (*p* < 0.05) (Table 3). Excessive polypharmacy was observed among ten (2.7%) and 14 (3.2%) participants for “polypharmacy 1” and “polypharmacy 2”, respectively.

#### 3.3.2. Factors Associated with Polypharmacy

Table 4 reports the results of the multivariable logistic regression. In the multivariable logistic regression model, multimorbidity (aOR=10.27; 95% CI: [3.97–26.57]) was the only factor associated with “polypharmacy 1”. Being female (aOR = 1.86; 95% CI: [1.00–3.47]) and having multimorbidity (aOR = 4.55; 95% CI: [2.42–8.54]) were the factors associated with polypharmacy 2.

## 4. Discussion

This study was conducted among 370 people aged 60 and over living in community dwellings of Lomé. The main reported medical conditions were osteoarthritis (59.5%), hypertension (50.5%) and diabetes (18.1%). More than two thirds of recruited elderly have reported self-medication practices and concurrent use of conventional drugs and medicinal plants and/or other dietary supplements was reported in almost 20% of study participants. The prevalence of polypharmacy (≥5 drugs) was 17.0% without medicinal plants and other dietary supplements, and it increased to 22.7% when medicinal plants and other dietary supplements were taken into account. Factors associated with polypharmacy were female sex and multimorbidity.

Polypharmacy is a public health threat, especially for the elderly among whom high prevalence of adverse drugs events have been described [2, 5]. Based on the threshold of five medications or more, polypharmacy was observed in one elderly in five in Lomé. Using the same quantitative definition, Tegegn et al., reported a polypharmacy prevalence of 19.7% among older patients visiting an outpatient clinic in Ethiopia [16]. Higher estimates were observed in Burkina Faso where almost three elderly in five (59.0%) were on polypharmacy [17]. However, comparisons between studies should be done with caution as the choice of the definitions of polypharmacy may affect the findings observed. Indeed, in the study conducted in Burkina Faso, polypharmacy was defined as the use of potentially inappropriate medications, based on the 2012 Beers criteria. There are numerous definitions of polypharmacy in the existing literature. Some researchers use a quantitative approach but there is no consensus on the threshold for defining polypharmacy [4, 5]. Another approach, based on qualitative assessment, is also widely used and polypharmacy is defined as “the inappropriate use of medications, including unnecessary drug use”, or use of suboptimal medications or therapeutic duplication [1, 4, 5, 8]. Hence, based on both qualitative and quantitative approaches, the prevalence of polypharmacy among elderly people varies greatly across studies, ranging between 5 and 78% [5].

In our study, the prevalence of polypharmacy increased by 5% when medicinal plants and other dietary supplements were included in the analyses. Also, concurrent use of conventional drugs and medicinal plants or other dietary supplements was observed among one in five elderly people in Lomé. In a systematic review, the prevalence of concurrent use ranged between 5.3 and 88.3% [6]. Medicinal plants are known to contain some bioactive compounds which can modify, either increasing or decreasing the activity of cytochromes P450 enzymes, thereby, modifying the bioavailability and depuration of drugs [26]. In a systematic review conducted in 2017, Agbabiaka et al., found that one in three concurrent users was at risk of a potential herb–drug or supplement–drug interaction [6]. Finally, the use of medicinal plants and other dietary supplements are associated with poor adherence to medications and more than 25% do not usually inform their physician regarding their use of these products [27]. Further studies are mandated on herb-drug interactions and polypharmacy in sub-Saharan Africa.

In multivariable analysis, multimorbidity and female gender were associated with polypharmacy. Our findings align with previous studies that described multimorbidity as a key factor of polypharmacy [28]. With ageing and the rise of chronic diseases such as diabetes, hypertension, and other cardiovascular diseases, elderly people are more likely to be on multiple therapeutic agents [28, 29]. Regarding gender, female sex was also identified as a factor associated with polypharmacy. This result is consistent with data reported in previous studies [30, 31]. Other factors such as the number of health care visits, higher body mass index, tobacco use, low income, lack of education, and self-medication have been reported in the literature as factors associated with polypharmacy [24, 29, 30]. However in our study, we did not collect data on health care visit, height, weight, and tobacco use to assess for these factors.

Although self-medication was not associated with polypharmacy in our study, it was highly prevalent among study participants (67.3%). This result is consistent with estimates reported among the elderly worldwide [12, 32–34]. Elderly people usually self-medicate because the symptomatology is familiar and known to them, delaying the time to visit a health professional. Also, in a study conducted in Iran, they stated being afraid of the announcement of bad news, when visiting a health professional [35]. Almost 40% of participants reported using drugs sold in the street for self-medication; this a concerning issue because these products could be contaminated with bacteria or be toxic with high levels of the wrong active ingredient or other toxic chemicals [36]. There is crucial need to organize health education campaigns highlighting the adverse effects of self-medication especially in elderly people in order to avoid severe and fatal outcomes.

This study has some limitations. We used the RDS method to recruit community dwelling elderly because this population is difficult to reach, specifically at home. However, we did not use the design effects to estimate the sample size and specific analyses to estimate medication consumption patterns [37]. The total number of drugs was recorded with no difference between prescribed and nonprescribed drugs. Therefore, the number of prescribed and nonprescribed drugs could not be assessed. Also, we did not include medication taken on monthly basis and this could lead to a classification bias because long-acting medication could have been missed. Finally, we did not assess the forms of consumption of medicinal plants (tablet, decoction) and future studies should document potential herb-drug interactions among the elderly. Nevertheless, this is the first study on medication consumption patterns among elderly people in Togo and we reported data on self-medication and polypharmacy. Also, it is one of the rare studies exploring polypharmacy in sub-Saharan African elderly living in the community, which has also assessed the use of medicinal plants and dietary supplements.

## 5. Conclusion

This first study on the medication consumption patterns of the elderly in Togo uncovered that almost seven elderly people in ten self-medicate and one older adult in five uses five or more medications daily. Togolese elderly also regularly used herbal products with around 20% of them reporting concurrent use of conventional drugs and medicinal plants. To reduce polypharmacy, there is a crucial need to sensitize elderly people on disease, and potential risk of self-medication, polypharmacy, and concurrent use. Further studies are needed to assess drug-drug interactions and herb-drug interactions among this population.

## Figures and Tables

**Table 1:** Socio-demographic and clinical characteristics of community-dwelling elderly in Lomé, Togo in 2017 (*N* = 370).

	Total (*N* = 370)	Male (*N* = 167)	Female (*N* = 203)	*p* value
*Age (years)*				
Median (IQR)	65 (62–71)	66 (63–69.5)	65 (62–73)	0.537^∗^

*Marital status, n (%)*				<0.001^∗∗^
Single	9 (2.4)	4 (2.4)	5 (2.5)	
Widowed	131 (35.4)	20 (12.0)	111 (54.6)	
Married/living with a partner	214 (57.8)	134 (80.2)	80 (39.4)	
Divorced	15 (4.1)	9 (5.4)	6 (3.0)	
MD	1 (0.3)	0 (0.0)	1 (0.5)	

*Education level, n (%)*				<0.001^∗∗^
None	100 (27.0)	17 (10.2)	83 (40.9)	
Primary school	107 (29.0)	42 (25.2)	65 (32.0)	
Secondary school	100 (27.0)	60 (35.9)	40 (19.7)	
University	60 (16.2)	47 (28.1)	13 (6.4)	
MD	3 (0.8)	1 (0.6)	2 (1.0)	

*Monthly revenue (euros), n (%)*				<0.001^∗∗∗^
<150	258 (69.7)	94 (56.3)	164 (80.8)	
(150–450)	77 (20.8)	55 (32.9)	22 (10.8)	
>450	20 (5.4)	12 (7.2)	8 (4.0)	
MD	15 (4.1)	6 (3.6)	9 (4.4)	

*Past medical history, n (%)*				
Hypertension	187 (50.5)	67 (40.1)	120 (59.1)	<0.001^∗∗∗^
Diabetes	67 (18.1)	30 (17.9)	37 (18.2)	0.948^∗∗∗^
Other CVD^1^	50 (13.5)	22 (13.2)	28 (13.8)	0.862^∗∗∗^
Osteoarthritis	219 (59.2)	84 (50.3)	135 (66.5)	0.002^∗∗∗^
Visual impairment^2^	46 (12.4)	20 (12.0)	26 (12.8)	0.809^∗∗∗^
Gastrointestinal disease^3^	32 (8.7)	18 (10.8)	14 (6.9)	0.186^∗∗∗^
Dyslipidemia	42 (11.4)	15 (8.9)	27 (13.3)	0.192^∗∗∗^

*Number of medical conditions, n (%)*				0.033^∗∗∗^
0	45 (12.2)	28 (16.8)	17 (8.4)	
1	112 (30.3)	54 (32.3)	58 (28.6)	
2	123 (33.2)	52 (31.1)	71 (35.0)	
≥3	90 (24.3)	33 (19.8)	47 (28.0)	

IQR=interquartile range, MD=Missing data. ^1^Other CVD= cardiovascular diseases including arrhythmia, cardiac conduction disorders, chronic congestive heart failure. ^2^Visual impairment including age-related degeneration, cataract, glaucoma, and blindness. ^3^Gastrointestinal diseases including peptic ulcer, hemorrhoids, chronic constipation, and chronic hepatitis. ^∗^Mann-Whitney *U* test; ^∗∗^Fisher's exact test; ^∗∗∗^Chi square test.

**Table 2:** Medications consumption patterns of community-dwelling elderly in Lomé, Togo in 2017 (*N* = 370).

	Total (*N* = 370)	Male (*N* = 167)	Female (*N* = 203)	*p* value
*Number of medications taken^1^*				0.074^∗^
Median (IQR)	2 (1–4)	2 (1–4)	3 (1–5)	

*Medications taken^1^, n (%)*				0.197^∗∗^
0	69 (18.6)	37 (22.2)	32 (15.8)	
1	51 (13.8)	21 (12.6)	30 (14.8)	
2	69 (18.6)	36 (21.5)	33 (16.2)	
≥3	179 (48.4)	72 (43.1)	107 (52.7)	
MD	2 (0.6)	1 (0.6)	1 (0.5)	

*Type of medication, n (%)*				
Conventional drug	290 (78.4)	127 (76.1)	163 (80.3)	0.083^∗∗∗^
Dietary supplement	35 (9.5)	16 (9.6)	19 (9.4)	0.124^∗∗∗^
Plants/Herbs	53 (14.3)	24 (14.4)	29 (14.3)	0.983^∗∗∗^

*Self-medication, n (%)*				0.518^∗∗^
Yes	249 (67.3)	116 (69.5)	133 (65.5)	
No	112 (30.3)	46 (27.6)	66 (32.5)	
MD	9 (2.4)	5 (2.9)	4 (2.0)	

*Concurrent use, n (%)*				
Yes	63 (17.0)	29 (17.4)	34 (16.7)	0.940^∗∗^
No	306 (82.7)	138 (82.6)	168 (82.8)	
MD	1 (0.3)	0 (0.0)	1 (0.5)	

IQR = interquartile range; MD = Missing data. ^1^Number of medications including medicinal herbs and dietary supplements. ^∗^Mann-Whitney *U* test; ^∗∗^Fisher's exact test; ^∗∗∗^Chi square test.

**Table 3:** Polypharmacy among community-dwelling elderly in Lomé, Togo in 2017.

	Polypharmacy 1^∗^			Polypharmacy 2^∗∗^		
	*n*/*N*	%	*p*	*n*/*N*	%	*p*
*Overall prevalence*	63/369	17.1		84/368	22.8	
*Sex*			0.210			0.049
Male	24/167	14.4		30/166	18.1	
Female	39/202	19.3		54/202	26.7	

*Age*			0.434			0.906
≤65 years	23/151	15.2		34/151	22.5	
>65 years	40/218	18.3		50/217	23.0	

*Marital status*			0.477			0.895
Married/Living with a partner	39/213	18.3		48/212	22.6	
Living alone	24/155	15.4		36/155	23.2	

*Educational level*			0.05			0.325
None	10/99	10.1		17/99	17.2	
Primary school	18/107	16.8		25/107	23.4	
Secondary school	25/100	25.0		28/99	28.3	
University	10/60	16.7		14/60	23.3	

*Monthly revenue*			0.188			0.654
<150€	39/257	15.2		55/256	21.5	
(150–450)	15/77	19.5		16/77	20.8	
>450€	6/20	30.0		6/20	30.0	

*Medical conditions*						
Hypertension	43/187	23.0	0.002	50/186	26.9	0.061
Other CVD	21/50	42.0	<0.001	26/50	52.0	<0.001
Diabetes	19/67	28.4	0.007	22/66	33.3	0.025
Osteoarthritis	49/218	22.5	0.001	61/217	28.1	0.004
Visual impairment	19/46	41.3	<0.001	21/46	45.7	<0.001
Dyslipidemia	17/42	40.5	<0.001	19/42	45.2	<0.001

*Multimorbidity*	58/213	27.2	<0.001	70/212	33.0	<0.001

^∗^Polypharmacy 1: number of medications ≥5, not including medicinal plants and other dietary supplements. ^∗∗^Polypharmacy 2: number of medications ≥5, including medicinal plants and other dietary supplements. CVD = cardiovascular disease.

**Table 4:** Multivariable analysis: factors associated with polypharmacy among community-dwelling elderly in Lomé, Togo, 2017.

	Polypharmacy 1^∗^			Polypharmacy 2^∗∗^		
	*n*/*N*	aOR (95% CI)	*P*	*n*/*N*	aOR (95% CI)	*p*
*Sex*			0.076			0.049
Male	24/167			30/166	1	
Female	39/202			54/202	1.86 [1.00–3.47]	

*Age*			0.279			0.847
≤65 years	23/151			34/151		
>65 years	40/218			50/217		

*Marital status*			0.146			
Married/Living with a partner	39/213			48/212		
Living alone	24/155			36/155		

*Educational level*			0.092			0.182
≤Primary	28/206			42/206		
>Primary	35/160			42/159		

*Multimorbidity*			<0.001			<0.001
No	5/156	1		14/156	1	
Yes	58/213	10.27 [3.97–26.57]		70/212	4.55 [2.42–8.54]	

^∗^Polypharmacy 1: number of medications ≥5, not including medicinal plants and other dietary supplements. ^∗∗^Polypharmacy 2: number of medications ≥5, including medicinal plants and other dietary supplements. 95% CI: 95% confidence interval; aOR: adjusted Odds Ratio.

## Data Availability

All data used for the present study are available and could be requested from the authors.

## Abbreviations

95% CI: 95% confidence interval
aOR: Adjusted Odds Ratio
CVD: Cardiovascular disease
IQR: Interquartile range
MD: Missing data.
